# Effects of Deposition Strategy and Preheating Temperature on Thermo-Mechanical Characteristics of Inconel 718 Super-Alloy Deposited on AISI 1045 Substrate Using a DED Process

**DOI:** 10.3390/ma14071794

**Published:** 2021-04-05

**Authors:** Ho Kim, Kwang-Kyu Lee, Dong-Gyu Ahn, Hyub Lee

**Affiliations:** 1 Department of Mechanical Engineering, Chosun University, Gwang-ju 61452, Korea; vvvrla@naver.com (H.K.); otq_center@naver.com (K.-K.L.); 2 Intelligent Manufacturing R&D Department, Korea Institute of Industrial Technology, Siheung-si 15014, Korea; leehyub@kitech.re.kr

**Keywords:** deposition strategy, preheating temperature, thermomechanical characteristics, Inconel 718 super-alloy, AISI 1045 substrate, DED process, finite element analysis

## Abstract

Thermomechanical characteristics are highly dependent on the deposition strategy of the directed energy deposition (DED) process, including the deposition path, the interpass time, the deposition volume, etc., as well as the preheating condition of the substrate. This paper aims to investigate the effects of the deposition strategy and the preheating temperature on thermomechanical characteristics of Inconel 718 super-alloy deposited on an AISI 1045 substrate using a DED process via finite element analyses (FEAs). FE models for different deposition strategies and preheating temperatures are created to examine the thermomechanical behavior. Sixteen deposition strategies are adopted to perform FEAs. The heat sink coefficient is estimated from a comparison of temperature histories of experiments and those of FEAs to obtain appropriate FE models. The influence of deposition strategies on residual stress distributions in the designed model for a small volume deposition is examined to determine feasible deposition strategies. In addition, the effects of the deposition strategy and the preheating temperature on residual stress distributions of the designed part for large volume deposition are investigated to predict a suitable deposition strategy of the DED head and appropriate preheating temperature of the substrate.

## 1. Introduction

Metal additive manufacturing (MAM) processes have been regarded as the cutting-edge technology leading the 4th industrial revolution [1,2]. Powder bed fusion (PBF) and directed energy deposition (DED) processes are representative MAM processes to fabricate three-dimensional metallic parts [3,4,5]. The DED process irradiates a focused energy source on the substrate to create the melt pool into the substrate [6,7,8]. The feeding materials are supplied into the melt pool. The feeding materials are melted by the heat of both the melt pool and the irradiated energy source [6,8]. The deposition bead is formed through the solidification of melted materials [6,7,8]. Three-dimensional metallic parts are fabricated by repetition of these steps. The DED process can be classified into powder and wire feeding types according to feeding materials [8,9]. The laser engineered net shaping (LENS) process is the first commercialized powder feeding type DED process by Optomec Inc. [8,9]. The LENS process has been patented by Sandia National Laboratory of USA [5,8]. The DED process has been widely applied to repair, remanufacturing and functional coating of metallic components [10,11]. In addition, the DED process can easily fabricate a functionally graded material (FGM) through selective deposition of dissimilar materials on the substrate [12,13]. The importance of the DED process has been greatly increased owing to the potential for developing a hybrid AM system [8,14,15]. Recently, various hybrid AM systems incorporated powder feeding type DED, and subtractive processes have widely used to fabricate metallic parts with complex shapes [8,16].

The quality and the failure of the deposited part by the DED process are significantly influenced by thermo-mechanical characteristics, including temperature and residual stress distributions, in the vicinity of the irradiated region by the heat source during deposition of the material on the substrate [17,18,19,20,21]. To prevent the occurrence of defects in the fabricated part by the DED process, it is necessary to examine the thermo-mechanical behavior of the boundary region between the deposited region and the substrate through analytical and experimental approaches [18,19]. Stender et al. proposed thermal-mechanical finite element (FE) flow for LENS process modeling [20]. They validated the FE model by comparing estimated temperature distributions by FEAs and measured temperature distributions by a forward-looking infrared (FLIR) camera. The thermo-mechanical characteristics are highly dependent on the deposition strategy of the DED process, including the deposition path, the interpass time, the deposition volume, etc., and the preheating condition of the substrate [21,22,23]. Ren et al. investigated the influence of the deposition path on thermal histories and residual stress distributions in the AISI 316 part to estimate the optimized path planning for a DED process using finite element analyses (FEAs) and experiments [24]. They adopted spiral, unidirectional and zig-zag paths for the deposition [24]. Lu et al. studied the effects of the building strategy on the thermo-mechanical response of Ti-6Al–4V rectangular parts manufactured by a DED process using FEAs [25]. They used six types of deposition path [25]. They also applied different paths to even and odd layers [25]. Li and Soshi simulated the formation of grain morphologies of the AISI 304 deposited region for unidirectional and bidirectional deposition patterns in a DED coating using a kinetic Monte Carlo (KMC) Potts model [26]. Saboori et al. examined the effects of the deposition path on the microstructure, the mechanical properties and the residual stress of the AISI 316L part produced by a DED process through experiments [27]. Ribeiro et al. performed an experimental investigation into the influence of the deposition path and the scan distance on the shape, the morphology, the surface roughness, the microstructure, and the microhardness of the AISI 316L deposited region by a DED process [28]. They used four deposition paths, including unidirectional directional, zig-zag, chessboard, and contour paths, and two scan distances [28]. Lu et al. investigated the effects of the deposition path and the preheating temperature on the distortion's evolution and the residual stress in a Ti-6Al–4V thin-wall structure fabricated by a DED process using FEAs [19]. Baek et al. carried out an experimental investigation of the effects of preheating of a D2 substrate on the microstructure, the hardness, the tensile strength, and the toughness of the M4 deposited part using a DED process [29]. They reported that an excessive preheating of the substrate causes the strength and the toughness to deteriorate [29]. Corbin et al. examined the influence of the substrate thickness and the preheating temperature on the distortion of the Ti-6Al–4V part deposited by a DED process [30]. They showed that the preheating could reduce the accumulated distortion of the deposited part by the DED process [30]. Lu et al. investigated the effects of preheating temperature and path on distortion histories and residual stress distributions of rectangular and S-shaped Ti–6Al–4V parts produced by a DED process using FEAs [31]. Soshi et al. proposed a trochoidal deposition path to increase the preheating temperature of the substrate in a DED process [32]. Zhao et al. examined the variation of the geometrical characterization, the interfacial microstructure and the mechanical property of specimens fabricated by a DED process using experiments [33]. They fabricated specimens through the deposition of AISI 316L steel on P20 and AISI 1045 steel substrates [33]. In the fabrication of heterogeneous materials, defects and distortion frequently occur due to differences in thermal and mechanical properties between the deposited material and the substrate. In particular, a thermomechanical investigation is necessary to successfully deposit super-alloy powders on a ferrous metal substrate without any defects in the fabricated part using the DED process. Although various previous research works have been carried out, the influence of the deposition strategy and the preheating conditions on the thermomechanical behavior to deposit Inconel super-alloy powders on the steel substrate using a DED process, however, has hardly been investigated to date.

The aim of this paper is to investigate the effects of the deposition strategy and the preheating temperature on thermomechanical characteristics of the Inconel 718 super-alloy deposited on the AISI 1045 substrate using a DED process via FEAs. FE models for different deposition strategies and preheating temperatures are created. Sixteen deposition strategies are adopted to perform FEAs. The coefficient of the heat sink is estimated from a comparison of the temperature history of experiments and that of FEAs to obtain appropriate FE models. The influence of the deposition strategy on residual stress distributions in the designed model for a small volume deposition is examined to determine feasible deposition strategies. In addition, the effects of the deposition strategy and the preheating temperature on residual stress distributions of the designed part for a large volume deposition are investigated to predict a suitable deposition strategy of the DED head and appropriate preheating temperature of the substrate.

## 2. Finite Element Analysis (FEA) and Experiments

### 2.1. FEAs

**Figure 1:**
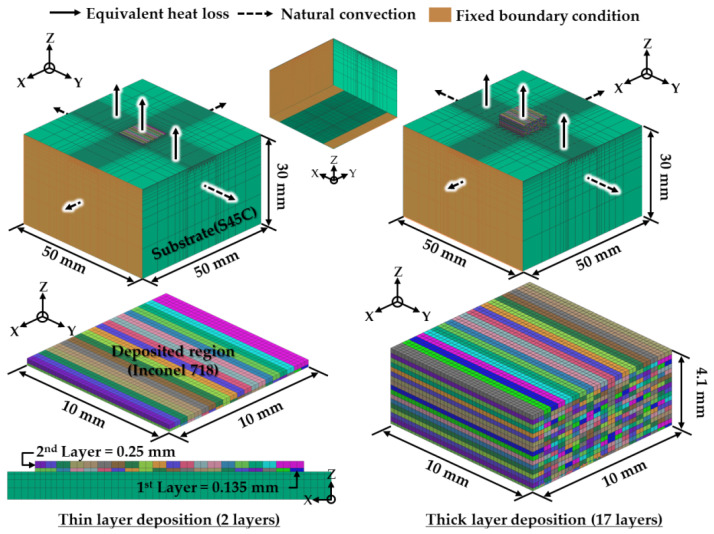
Models for finite element analyses (FEAs).

FEAs are carried out using the commercial software SYSWELD [34]. FE models for different deposition strategies are created, as shown in Figure 1. The dilution layer in the substrate is not considered in the FE models. It is also assumed that the deposited region is joined to the substrate. FE models with two layers are adopted to estimate feasible deposition strategies. In addition, FE models with 17 layers are employed for predicting a suitable heat sink coefficient, deposition strategy and preheating temperature. Dimensions of the substrate are 50 mm (length) × 50 mm (width) × 30 mm (height), as shown in Figure 1. In-plane dimensions of the deposited region are 10 mm (length) × 10 mm (width).

Table 1 shows characteristic dimensions of bead and deposition for FE models. The characteristic dimensions are obtained from results of repeated experiments of the 3D Printing Center of Korea Institute of Industrial Technology (KITECH) using a hybrid additive manufacturing system incorporated a LENS process. Deposition conditions for the characteristic dimensions are shown in Table 2. The radius (r_e_), the power (P_L_) and the scan speed (V) of the laser beam are set to 0.5 mm, 350 W and 1000 mm/min, respectively, as shown in Table 2. The feed rate of powders and the flow rate of shield gas are set to be nearly 10.3 g/min and 7.0 L/min, respectively. The distance between centers of successive beads, where porosity is minimized, is chosen as the hatch distance between successive beads. The initial width of the deposited bead and the hatching distance for a successive deposition is set to 0.78 mm and 0.5 mm, respectively. The thicknesses of beads for the first and second layers are set to 0.135 mm and 0.25 mm, respectively. The heights of the deposited region for 2 and 17 layer models are 0.385 mm and 4.135 mm, respectively. The initial width of the bead is applied to the first layer deposition, while that is not considered from the second layer to final layer depositions.

The laser beam of the DED process is assumed to be a three-dimensional volumetric heat flux with the penetration depth. The intensity distribution of the heat flux ($\dot{Q_{L}}$) is defined as Equation (1). $\bar{x}$ and $\bar{y}$ are given by Equations (2) and (3) [35]. Table 2 shows conditions of FEAs. In the FEAs, the penetration depth of the heat flux model is set to the bead thickness for each layer, as shown in Table 2. It is assumed that the radius of the laser beam at the *z* coordinate in the penetration direction is almost the same as the effective radius of the laser beam at the top surface, as shown in Table 2. The heat flux is applied to deposited regions during the deposition:

(1) $$\dot{Q_{L}}=\frac{P_{L}}{\pi \delta r_{e}^{2}V}\exp\left(-\frac{{\bar{x}}^{2}+{\bar{y}}^{2}}{16r{\left(z\right)}^{2}}\right)\;for\;r\leq r\left(z\right)\;and\;z_{p}\leq z\leq z_{e}$$

where $\dot{Q_{L}}$, η, P_L_, δ, r_e_, V, $\bar{x}$, $\bar{y}$, *r*(*z*), *r*, *z*, *z*_p_, and *z*_e_ are the linear intensity of the heat flux, the efficiency of the laser beam, the power of the laser beam, the penetration depth of the laser beam, the effective radius of the laser beam, the scan speed of the laser beam the moving coordinate in the *x*-direction, the moving coordinate in the *y*-direction, the radial distance from the center of the laser beam, the z-coordinate at a distance δ from the surface of the bead, and the z-coordinate at the surface of the bead, respectively [2].

(2) $$\bar{x}=x-V\cdot t\;\mathrm{for}\;\mathrm{the}\;\mathrm{deposition}\;\mathrm{in}\;\mathrm{the}\;\mathrm{x-direction}$$

(3) $$\bar{y}=y-V\cdot t\;\mathrm{for}\;\mathrm{the}\;\mathrm{deposition}\;\mathrm{in}\;\mathrm{the}\;\mathrm{y-direction}$$

where *x*, *y*, *V* and *t* are the *x* coordinate, the y coordinate, the scan speed of the laser beam and the time, respectively.

**Figure 2:**
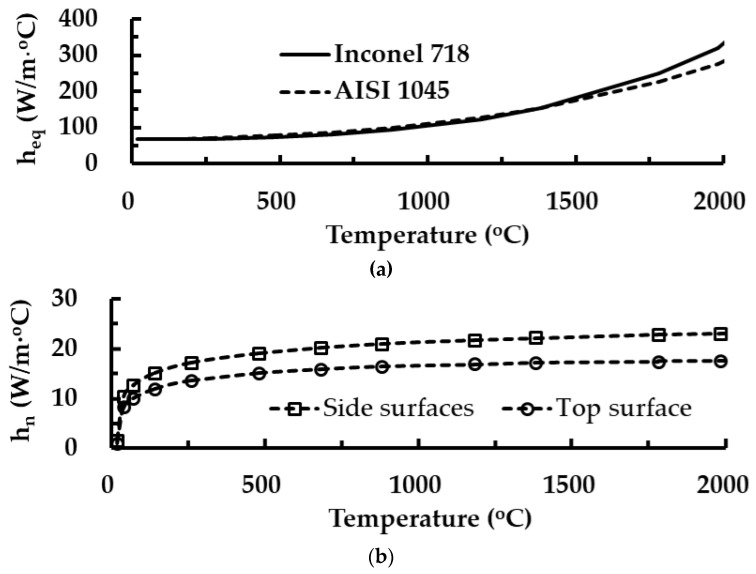
Coefficients of applied boundary conditions for heat transfer analyses: (**a**) temperature-dependent coefficients of the equivalent heat loss model (*h*_eq_); (**b**) temperature-dependent coefficients of the natural convection (*h*_n_).

The equivalent heat loss model is applied to the top surface of the substrate and the deposited bead during the deposition, as shown in Figure 1 [23]. The equivalent heat loss model includes the forced convection and the radiation terms together, as shown in Equations (4) and (5) [23]. Figure 2a shows the estimated coefficient of the equivalent heat loss model. The contacted region between the substrate and the fixture for the experiment is assumed to be a heat sink, as shown in Equation (6) [35]. The coefficient of the heat sink model is predicted by the comparison of results of experiments with those of FEAs from the viewpoint of temperature histories for multilayer deposition. The natural convection condition with temperature-dependent convection coefficients is applied to boundary surfaces of the substrate exclusive of application surfaces of the equivalent heat loss and the heat sink during the deposition, as shown in Figure 1. Figure 2b shows the predicted temperature-dependent natural convection coefficient (*h*_n_). The natural convection is applied to all surfaces during cooling after deposition:

(4) $${\dot{Q}}_{eq}=h_{eq}A\left(T_{s}-T_{a}\right)$$

where ${\dot{Q}}_{eq}$, $h_{eq}$, *A*, *T*_s_ and *T*_a_ are the equivalent heat loss, the equivalent heat loss coefficient, the surface area, the surface temperature, and the environment temperature, respectively:

(5) $$h_{eq}=h{\left(T\right)}_{fo}+\varepsilon \sigma \left(T_{s}^{2}+T_{a}^{2}\right)\left(T_{s}+T_{a}\right)$$

where $h_{fo}$, *ε* and *σ* are the forced convection coefficient, the emissivity and Boltzmann–Stefan constant, respectively:

(6) $${\dot{Q}}_{hs}=h_{s}A_{s}\left(T_{ss}-T_{a}\right)$$

where ${\dot{Q}}_{hs}$, $h_{s}$, *A_s_*, and *T_ss_* are the heat loss in the contacted surface, the heat sink coefficient, the contact area, the temperature of the contact surface, respectively.

The fixed boundary condition is applied to the side and bottom surfaces of the substrate in contact with the fixture of experiments during the deposition, as shown in Figure 1. The fixed boundary condition is applied to the center region of the substrate during the cooling stage. The residual stress is predicted by an algorithm of SYSWELD software [36]. The elastoplasticity is used to estimate residual stress distributions. The total strain increment is calculated by the summation of elastic, plastic and thermal strain increments. The Von-Mises criterion is used to evaluate yielding. The isotropic strain hardening model is adopted to estimate an equivalent strain and the corresponding flow stress.

**Figure 3:**
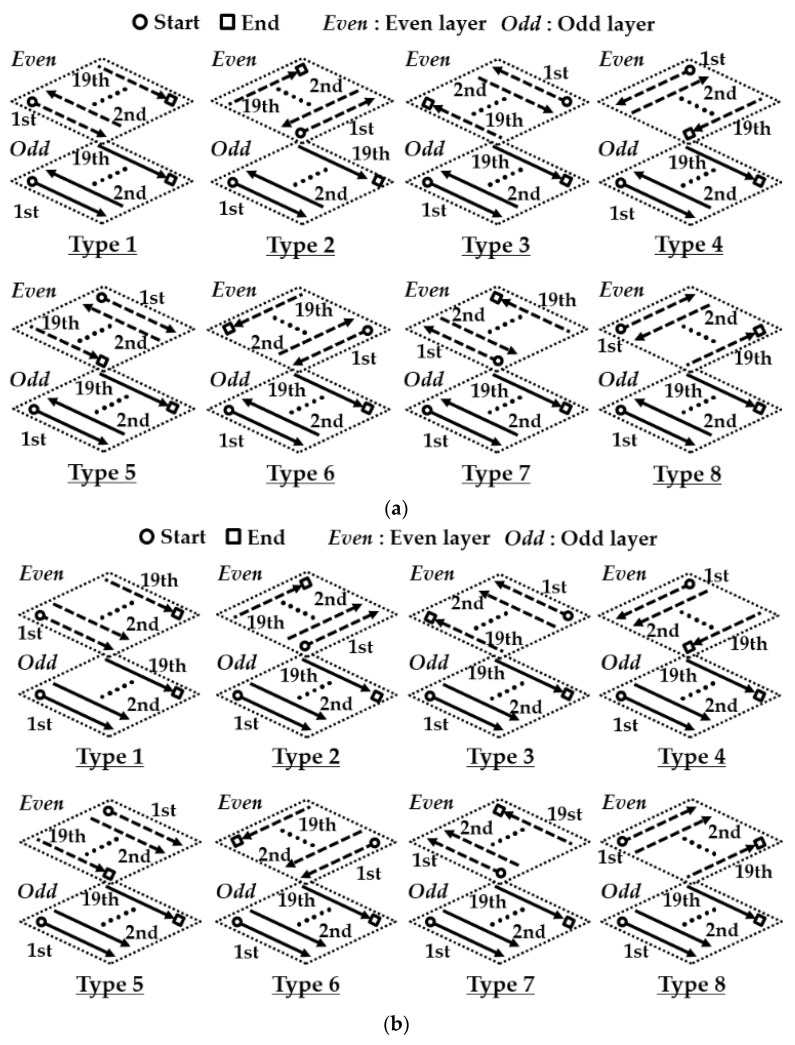
Deposition strategies for FEAs: (**a**) alternative directional deposition strategies (ADDSs); (**b**) uni-directional deposition strategies (UDDSs).

Sixteen deposition strategies are adopted to carry out FEAs, as shown in Figure 3. The deposition strategy fundamentally consists of alternative directional and unidirectional deposition paths. Identical deposition direction and starting point of the DED head are applied to the odd layer. The deposition direction and the starting point are changed for the case of the even layer. Eight types of deposition paths are contrived according to the combination of the deposition path and the starting point for the case of the even layer. The preheating temperature of the substrate ranges from 100 °C to 200 °C, as shown in Table 2.

**Figure 4:**
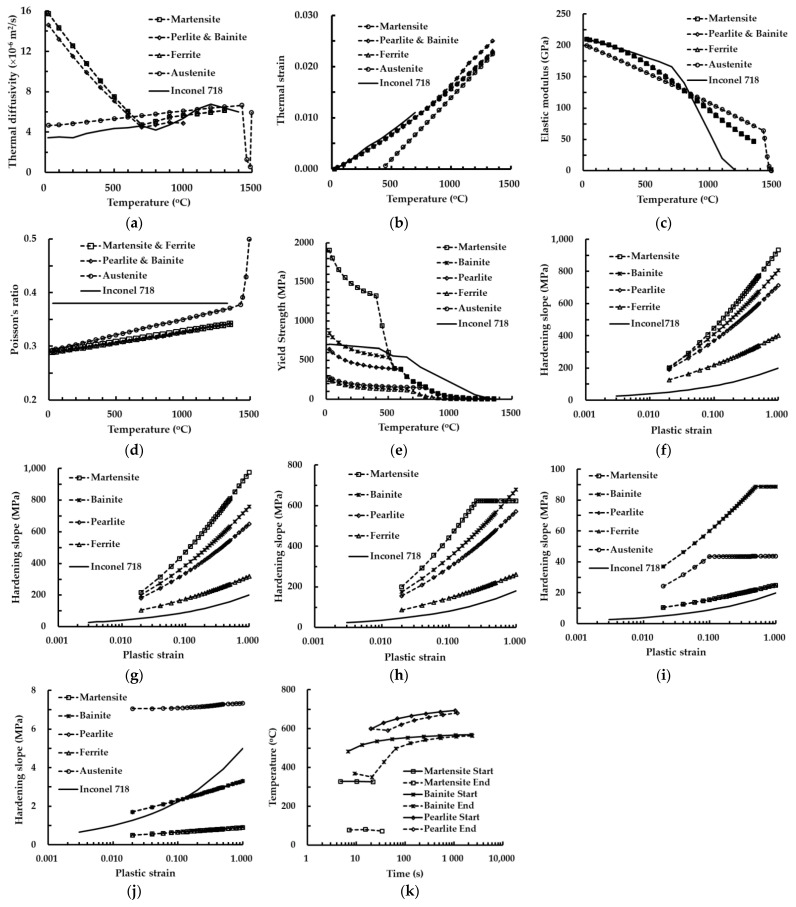
Thermal and mechanical properties of Inconel 718 and AISI 1045 [34,38]: (**a**) thermal diffusivity; (**b**) thermal strain; (**c**) elastic modulus; (**d**) Poisson’s ratio; (**e**) yield strength; (**f**) hardening slope—plastic strain curve (room temperature); (**g**) hardening slope—plastic strain curve (temperature = 200 °C); (**h**) hardening slope—plastic strain curve (temperature = 400 °C); (i) hardening slope—plastic strain curve (temperature = 800 °C); (**j**) hardening slope—plastic strain curve (temperature = 1200 °C); (**k**) continuous cooling transformation (CCT) diagram of AISI 1045.

Inconel 718 and AISI 1045 are chosen as deposition and substrate materials, respectively. Inconel 718 has widely applied to aircraft engines, rocket, nuclear parts due to excellent properties in the elevated temperature [37]. AISI 1045 structural steel has commonly used to fabricate shaft, gear, mold, die, pin, etc. Temperature-dependent thermal and mechanical properties are used to perform FEAs, as shown in Figure 4. Inconel 718 is assumed to be a single-phase material with a gamma phase. Thermal and mechanical properties of all phases are used to perform the FEAs for the case of AISI 1045, as shown in Figure 4. Thermomechanical properties of AISI 1045 are predicted by JMat Pro software [38]. Thermal and mechanical properties of the SYSWLED database are used for the case of Inconel 718 [34]. Hardening slope-plastic strain curves for different specimen temperatures are analytically estimated by JMat Pro software [38]. The necking phenomenon and the fracture of the specimen are not considered in the estimated hardening slope-plastic strain curves. Inconel 718 and AISI 1045 are assumed as isotropic materials. The initial phase fraction of the substrate is set to be 25% ferrite and 75% pearlite using JMat Pro data [38].

### 2.2. Experiments

**Figure 5:**
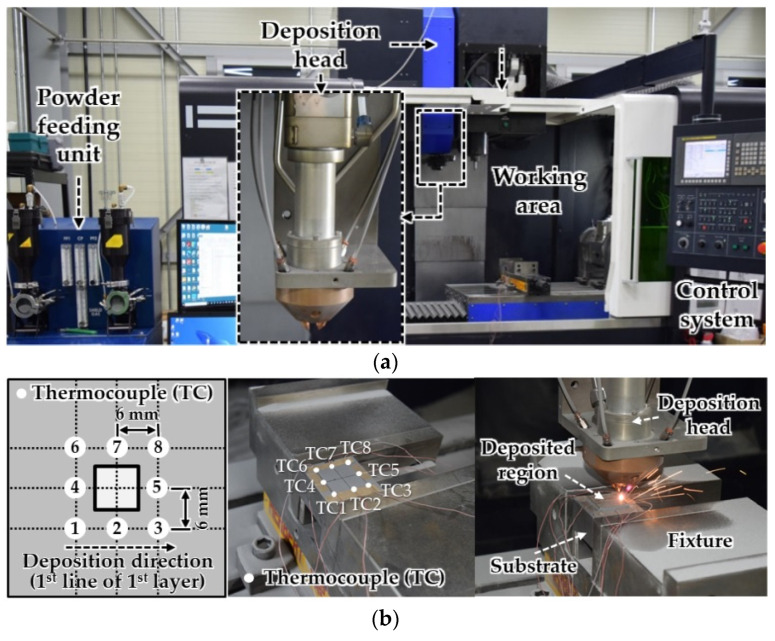
Experimental methodology: (**a**) hybrid directed energy deposition (DED) system incorporated a laser engineered net shaping (LENS) process; (**b**) experimental setup.

To measure temperature histories in the specimen during the deposition, deposition experiments are carried out using a hybrid DED system of 3D Printing Center of Korea Institute of Industrial Technology, as shown in Figure 5a. The hybrid DED system incorporates a LENS process of Optomec Inc., as shown in Figure 5a. Table 2 shows the conditions of the deposition experiments. Inconel 718 powders of VDM Metals Inc. in GmbH are adopted to perform the deposition experiment [39]. The diameter of Inconel 718 powder particles ranges from 53 µm to 100 µm [39]. AISI 1045 structural steel plate of POSCO Inc. is used as the substrate. Table 3 and Table 4 show chemical compositions of used Inconel 718 powders and AISI 1045 structural steel, respectively. The sand-blasting process is applied to the substrate as preprocessing before deposition experiments.

Eight thermocouples (TCs) are attached to the top surface of the substrate, as shown in Figure 5b. A J-type thermocouple of OMEGA Inc. is used to measure temperatures at different locations of the specimen. Two deposition strategies, type 2 and 5 paths of alternative directional deposition strategies (ADDSs) are applied to the deposition experiment. The first deposited line is always created by the deposition of Inconel 718 powders from the edge near TC1 (starting point) to the edge near TC3. Installation locations of thermocouples on the substrate are indicated in Figure 5b. Three deposition experiments are carried out for each deposition strategy. The temperature history for each deposition strategy is estimated from the average of the results of the experiments.

## 3. Results and Discussion

### 3.1. Coefficient of Heat Sink

**Figure 6:**
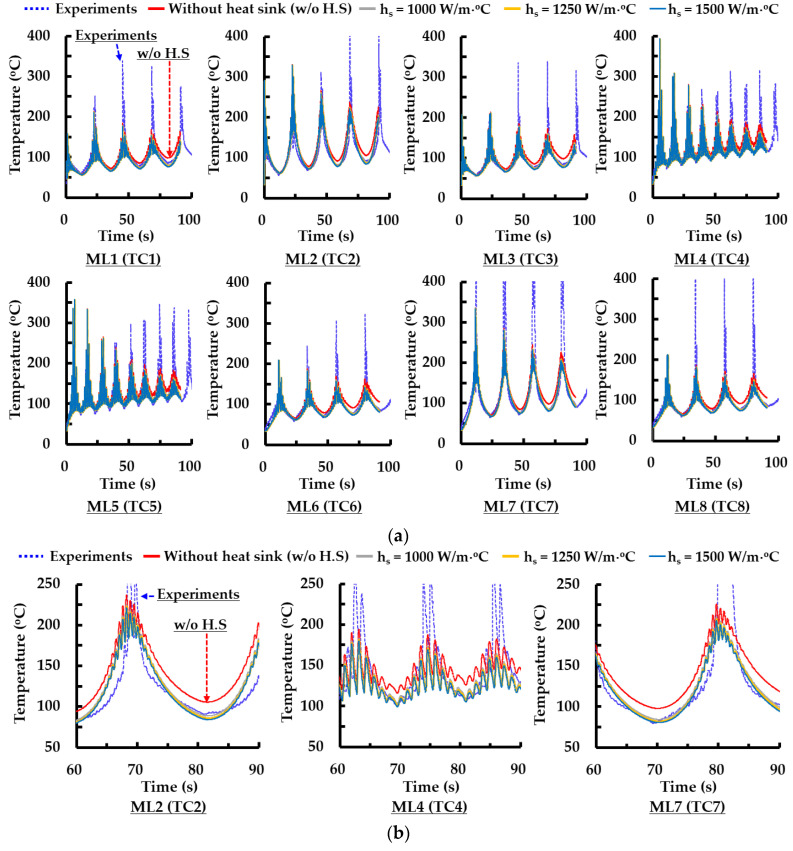
Comparison of temperature histories of experiments and those of FEAs for different coefficients of the heat sink (type 5 path of ADDSs): (**a**) temperature histories for all measured locations (from the first layer deposition to the 8th layer deposition); (**b**) detailed temperature histories for selected deposition times and measured locations (MLs).

**Figure 7:**
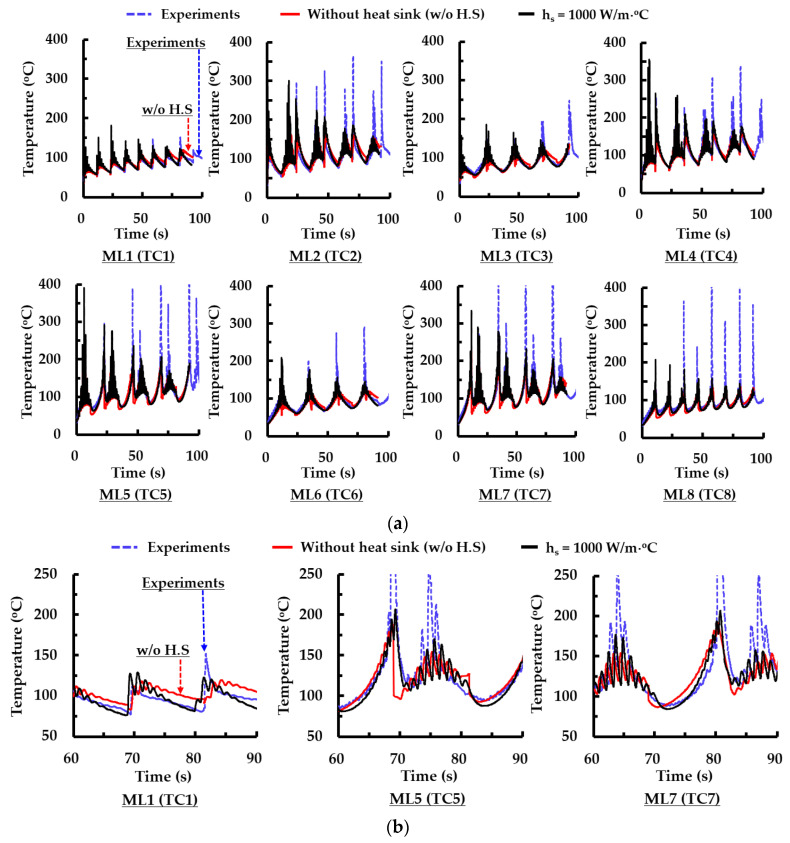
Comparison of temperature histories of experiments and those of FEAs for different heat sink conditions (type 2 path of ADDSs): (**a**) temperature histories for all measured locations (from the first layer deposition to the 8th layer deposition); (**b**) detailed temperature histories for selected deposition times and MLs.

To estimate a suitable coefficient of the heat sink, the results of FEAs are compared to those of experiments in terms of temperature histories for different measurement locations. Figure 6 and Figure 7 show temperature histories for different measured locations from the first layer deposition to the 8th layer deposition. The coefficient of the heat sink ranges from 1000 W/m °C to 1500 W/m °C, as shown in Figure 6. The difference between measured and predicted temperatures increases with an increasing number of deposited layers regardless of deposition strategies when the heat sink is not applied to the FE model, as shown in Figure 6 and Figure 7.

Unlike the results of the deposition experiments, estimated temperatures by the FEA at measured locations (MLs) significantly increase during successive deposition of additional layers when the heat sink is not applied to the FE model. Estimated temperature histories by the FEAs are similar to the measured temperature histories by the TCs exclusive of the deposition time corresponding to a sudden temperature increase when the heat sink is applied to the FE model, as shown in Figure 6b and Figure 7b. From the observation of the deposition process, it is noted that the abrupt increase in the measured temperature by the TCs is caused by the occurrence of spatter when the DED head passes through the vicinity of the corresponding TCs, as shown in Figure 6 and Figure 7. When the DED head passes through the vicinity of measuring locations of thermocouples, many spatters fly around corresponding thermocouples. An amount of spatter augments when the number of deposition layers increases. Due to these phenomena, the temperature measured by the thermocouple abruptly increases when the DED head goes through the vicinity of the thermocouple.

Temperature histories estimated by the FEAs are hardly changed for the case of the type 5 path of ADDSs when the coefficient of the heat sink is varied from 1000 W/m °C to 1500 W/m °C, as shown in Figure 6. From this result, it is revealed that the value of the coefficient of the heat sink hardly affects temperature histories for different MLs in the range of the adopted coefficient for the FEAs. 

Based on the above results, a suitable coefficient of the heat sink is chosen as 1000 W/m °C. 

**Figure 8:**
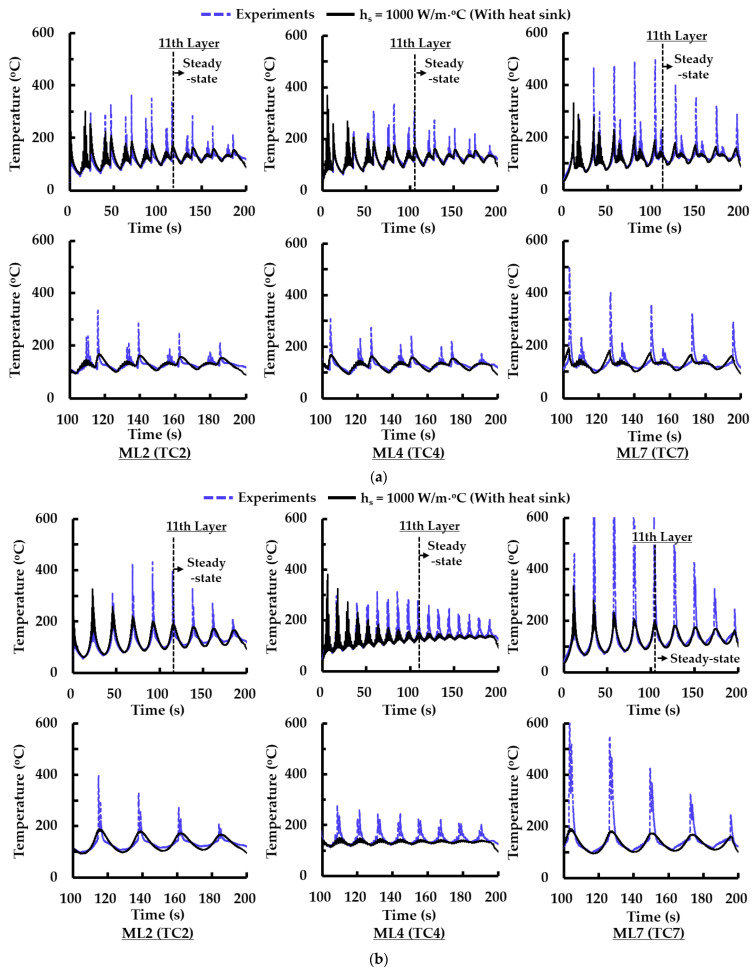
Comparison of temperature histories of experiments and those of FEAs for a large volume deposition (deposition of seventeen layers): (**a**) type 2 path for ADDS; (**b**) type 5 path for ADDS.

Figure 7 shows that temperature histories for the type 2 path of ADDSs can be properly predicted by FEAs when the selected suitable coefficient of the heat sink is applied to the FE model. From this result, it is revealed that the selected suitable coefficient of the heat sink is applicable to FE models for the other deposition strategies. Estimated temperature histories by FEAs, including the selected suitable coefficient of the heat sink, are compared to those measured by experiments for the case of type 2 and 5 paths of ADDSs when the number of deposited layers is totally seventeen layers as shown in Figure 8a,b. This figure shows that temperature histories estimated by the FEAs are fairly similar to those measured by experiments exclusive of the deposition time corresponding to a sudden temperature increase. From this result, it is shown that the FE model, including the selected suitable coefficient of the heat flux, can appropriately simulate a thermomechanical behavior of the designed model during the deposition. In addition, it is revealed that the FE model can be applicable to the simulation of thermomechanical characteristics of the designed model for thickly layered deposition. Temperatures for different MLs converge to specific temperatures from the 11th layer. From this result, it is noted that a steady-state heat transfer phenomenon appears from the deposition of the 11th layer.

### 3.2. Residual Stress Distributions and Feasible Deposition Strategies (FDSs)

**Figure 9:**
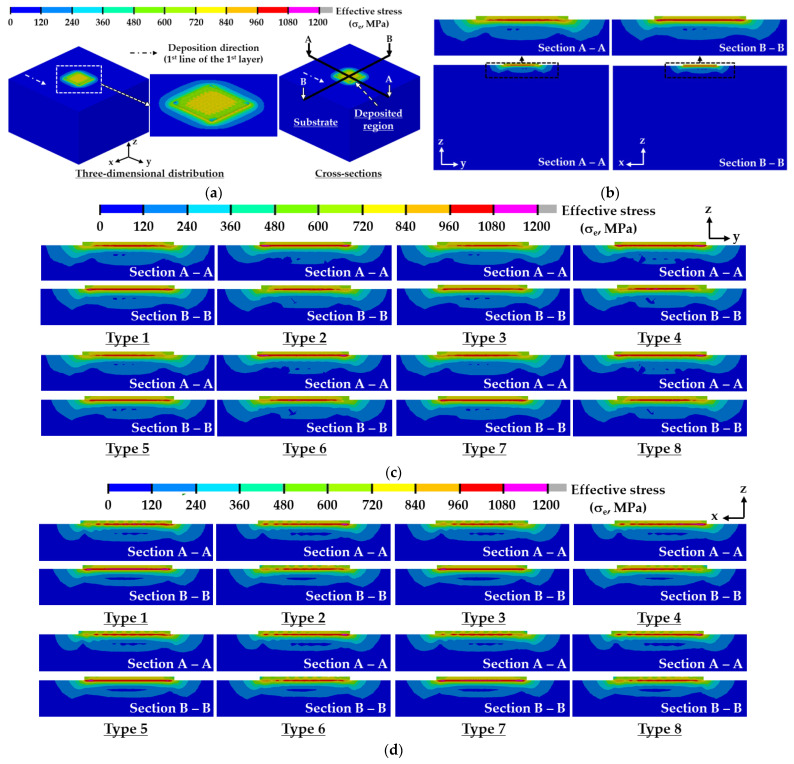
Effects of deposition strategies on effective stress distributions after the completion of cooling for a small volume deposition: (**a**) Three-dimensional distributions for type 5 path of ADDSs and cross-sections for residual stress distributions in the TD; (**b**) regions to estimate cross-sectional distributions of effective stress; (**c**) effective stress distributions in the TD (ADDS); (**d**) effective stress distributions in the TD (UDDS).

**Figure 10:**
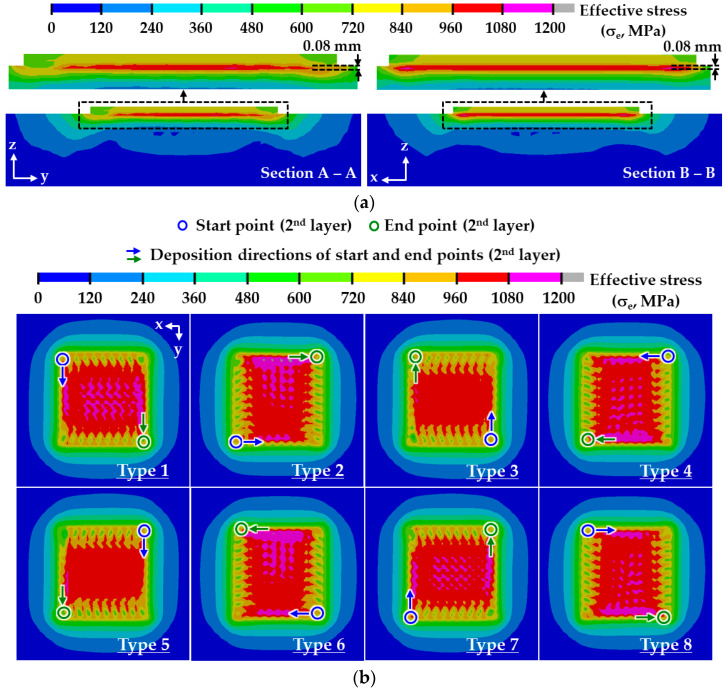

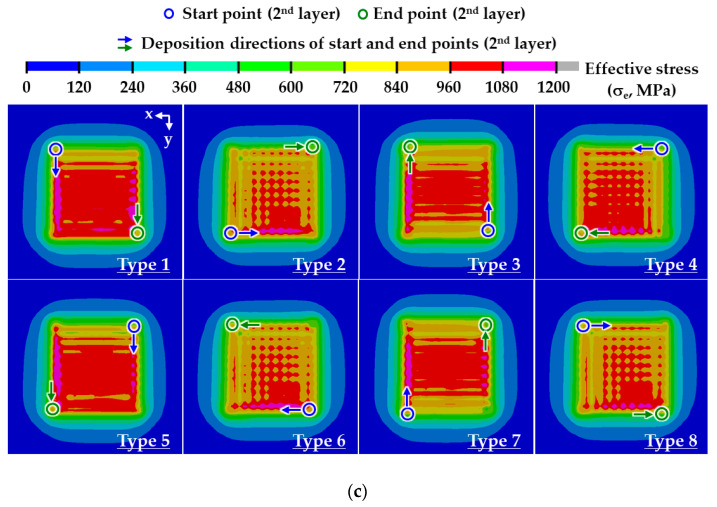
Effects of deposition strategies on planar effective stress distributions after the completion of cooling for a small volume deposition: (**a**) location of the plane to estimate effective stress distributions in the TD (type 1 path of ADDSs); (**b**) planar effective stress distributions (ADDS); (**c**) planar effective stress distributions (UDDS).

Figure 9 and Figure 10 show the effects of deposition strategies on effective stress distributions in the vicinity of the deposited region after the completion of the cooling stage for the case of a small volume deposition with two layers. To investigate the inside distribution of the residual stress, cross-sectional effective stress distributions for different deposition strategies are estimated, as shown in Figure 9c,d. The residual stress rapidly changes in the vicinity of the deposited region. Significant changes in the residual stress are observed in the vicinity of the deposited region. An excessive effective stress distribution appears in the vicinity of the boundary between the deposited region and the substrate, irrespective of the deposition strategies. Most of the regions where excessive residual stress occurs are observed in the top region of the substrate below the deposited bead regardless of the deposition strategies. The depth of the excessively stressed region (ESR) is less than 200 µm. The cross-sectional residual stress distribution of the ESRs for ADDSs is significantly different from that for unidirectional deposition strategies (UDDSs). The cross-sectional residual stress distribution of the ESRs forms almost symmetrically concerning the centerline of the deposited bead for ADDSs, while that appears asymmetrically for UDDSs. The thickness of the ESRs is nearly constant for ADDSs, while that partially changes for UDDSs.

The planar residual stress distribution is estimated from a plane, including the first node of the substrate in the depth direction from the top surface of the substrate, as shown in Figure 10a. The distance from the top surface of the substrate to the plane for prediction of the planar distribution of the effective stress is nearly 0.08 mm. Figure 10b,c show planar effective stress distributions for different deposition strategies. The ESR in the defined plane for the ADDSs shows a rectangular shape with two axes of symmetry irrespective of types of deposition path, as shown in Figure 10b. In the case of the ADDSs, the deposition path of the second layer for type 1, 3, 5, and 7 paths is perpendicular to that of types 2, 4, 6, and 8, as shown in Figure 3a. The long side of the ESR in the defined plane is formed in the perpendicular direction to the deposition direction of the 2nd layer when ADDSs are adopted, as shown in Figure 10b. The planar distribution of the residual stress is greatly influenced by the angle between successive layers for the case of ADDSs.

Unlike the ADDSs, the planar shape of the ESR for the UDDSs is predicted to be an asymmetrical shape, as shown in Figure 10c. The planar effective stress distribution of the ESR for the ADDSs is almost uniform, while that for the UDDSs is somewhat irregular. A significant imbalance of the residual stress appears for the case of the UDDSs, as shown in Figure 10c. The area of the ESR for UDDSs is significantly smaller than that for the ADDSs. From these results, it is considered that the possibility of the failure and distortion of the deposited part increases when UDDSs are applied. Unlike ADDSs, the planar residual stress distribution is significantly changed according to the type of the deposition path for the case of UDDSs. From this result, it is noted that the deposition direction and the angle between successive layers greatly affect the residual stress distribution in the ESR for the case of UDDSs.

**Figure 11:**
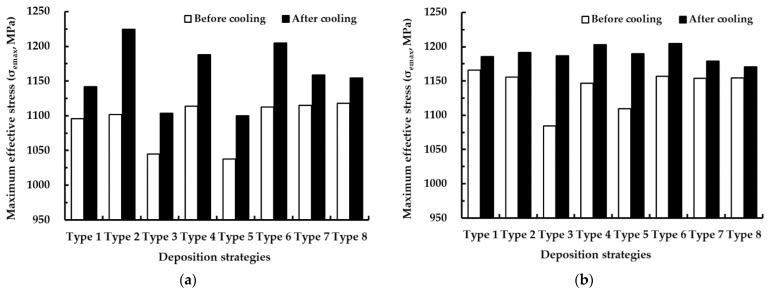
Effects of deposition strategies on maximum effective stresses for a small volume deposition: (**a**) ADDSs; (**b**) UDDSs.

The maximum residual stress appears in the vicinity of the boundary of the ESR irrespective of deposition strategies, as shown in Figure 10b,c. From this result, it is revealed that the failure always takes place in the vicinity of the boundary of the ESR. The critical location, where the maximum residual stress appears, is changed according to the deposition strategy. From this result, it is noted that the combination of the deposition direction and angle between successive layers greatly affects the critical location. Figure 11 shows the effects of deposition strategies on the maximum effective stresses in the FE model with a small deposition volume. The maximum effective stress after the completion of cooling is greater than that before cooling by nearly 1.4–11.2%. From this result, it is revealed that the natural cooling in the cooling stage and the elastic recovery induced by unclamping the fixed boundary increase the residual stress in the deposited part for the case of a small volume deposition. The mean value of the maximum effective stress after the completion of cooling for ADDSs is nearly 1160 MPa, while that for UDDSs is nearly 1189 MPa. This is because the area of the ESR for ADDSs is larger than that for UDDSs. The maximum effective stress after the completion of cooling for ADDSs exclusive of type 2 and 6 paths is less than that for UDDSs. From these results, it is shown that the residual stress in the deposited part can be reduced when the ADDSs are adopted. The maximum effective stress after the completion of cooling is reduced to nearly 1100 MPa for cases when type 3 and 5 paths of ADDSs are applied, as shown in Figure 11a. In addition, the maximum effective stress after the completion of cooling for UDDSs is minimized when the type 8 path is adopted, as shown in Figure 11b. From the results of FEAs for a small volume deposition, type 3 and 5 paths of ADDSs and type 8 paths of UDDSs are chosen as feasible deposition strategies (FDSs).

### 3.3. Thermo-Mechanical Characteristics and Suitable Deposition Strategy

**Figure 12:**
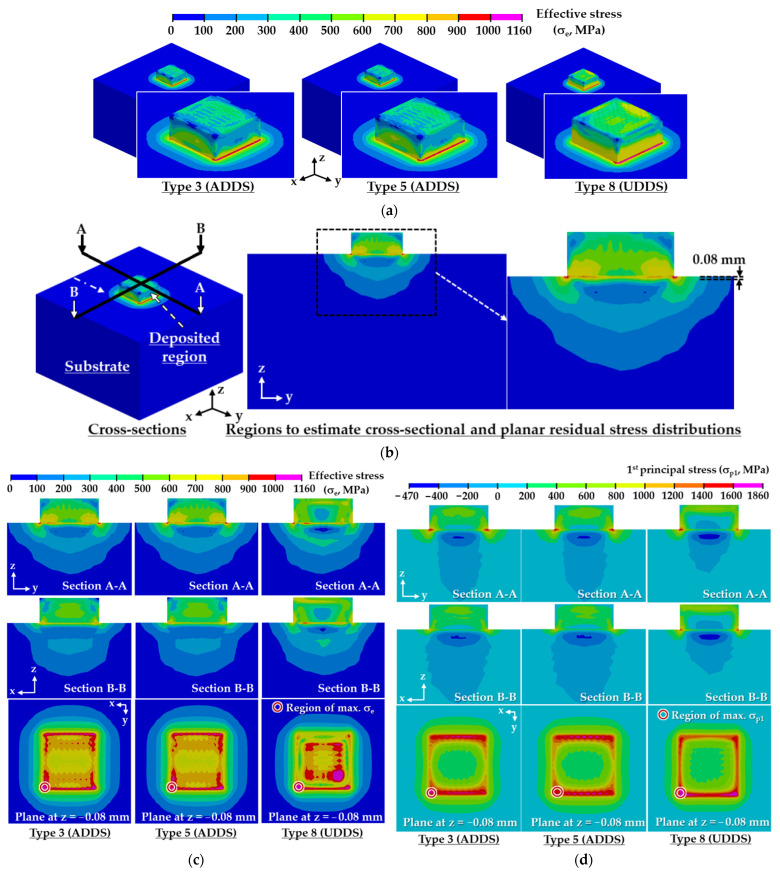

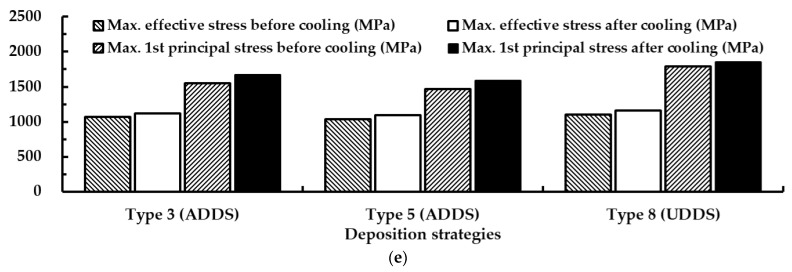
Influence of deposition strategies on residual stress distributions after the completion of cooling for a large volume deposition: (**a**) three-dimensional effective stress distribution for different FDSs; (**b**) regions to estimate cross-sectional and planar residual stress distributions; (**c**) cross-sectional and planar effective stress distributions for different FDSs; (**d**) cross-sectional and planar first principal stress (σ_p1_) distributions for different FDSs; (**e**) maximum residual stresses for different FDSs.

FEAs are performed for the case of a large volume deposition with 17 layers using predicted FDSs. Figure 12 shows residual stress distributions for different FDSs. Unlike a small volume deposition with two layers, the maximum residual stress appears in the vicinity of edges of the boundary between the deposited region and the substrate irrespective of FDSs for the case of a large volume deposition. From this result, it is shown that cracking takes place in the vicinity of the edges of the boundary between the deposited region and the substrate for the case of a large volume deposition.

The residual stress of the deposited region increases for the case of type 8 paths of UDDS than type 3 and 5 paths of ADDS, as shown in Figure 12a,c,d. Cross-sectional and planar distributions of the residual stress for ADDS are somewhat different from those for UDDS, as shown in Figure 12c,d. Unlike the UDDS, effective stress distributions of the ADDS appear almost symmetrical to reference axes of the model. The location of the maximum effective stress is identical to that of the maximum first principal stress. The high residual stress, which is marked in red and magenta colors in Figure 12c,d, is concentrated in a relatively small area for the case of the UDDS than the ADDS. The results of FEAs show that the region of maximum residual stress for the feasible deposition paths after the completion of cooling is found in an almost similar region, as shown in Figure 12c,d. This is attributed that imposed boundary conditions to the FE model during the elastic recovery greatly affects the formation of the residual stress in the deposited part after the completion of cooling.

**Figure 13:**
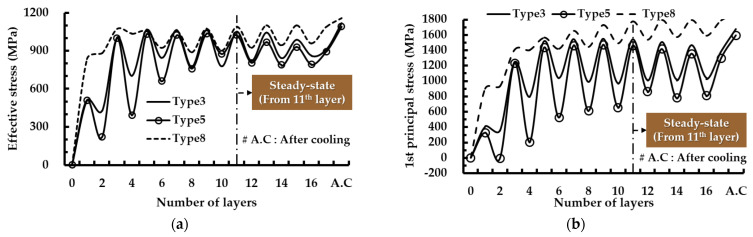
Histories of residual stresses at the location of maximum stress for different FDSs (large volume deposition): (**a**) effective stress; (**b**) first principal stress.

Figure 13 shows histories of residual stresses, including effective and first principal stresses, at the location of the maximum stress after completion of cooling for different FDSs. The effective and first principal stresses for each layer are obtained when the deposition of the corresponding layer is completed. The residual stress of type 8 paths for UDDS is greater than that of type 3 and 5 paths for ADDS, as shown in Figure 12e and Figure 13.

**Figure 14:**
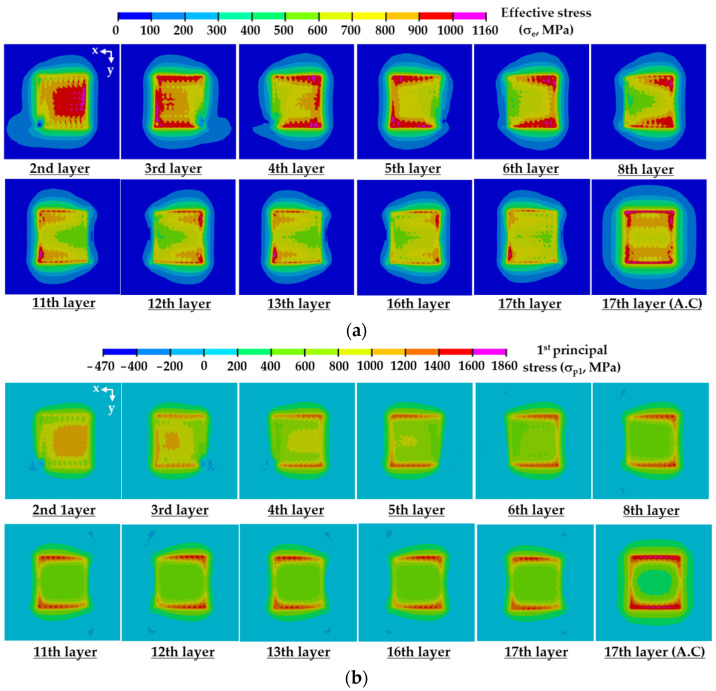
Residual stress distributions after deposition of each layer (large volume deposition, type 5 path of ADDS): (**a**) effective stress distributions; (**b**) first principal stress distributions.

The residual stress at the location of the maximum stress increases by nearly 4–8% after the completion of cooling. A highly stressed area significantly augments after completion of cooling, as shown in Figure 14. This is because the elastic recovery induced by unclamping of the fixed region during the cooling stage augments the residual stress in the vicinity of edges of the boundary between the deposited region and the substrate by increasing the relative deformation of the substrate and the deposited region. The formation of the residual stress changes from asymmetrical to symmetrical distributions via the elastic recovery induced by unclamping of the fixed boundary for the case of ADDSs. From these results, it is shown that the elastic recovery induced by unclamping during the cooling stage greatly affects the distribution and the value of the residual stress. Maximum effective and first principal stresses after the completion of cooling for UDDS are greater than those for ADDS by nearly 4% and 11%, respectively, as shown in Figure 12e. From this result, it is noted that the type 3 and 5 paths of ADDS prefer the type 8 path of UDDS from the viewpoint of safety. The residual stress at the location of the maximum stress increases with an increasing number of deposited layers for the case of the type 8 path of UDDS, while that almost converges to a specific value from the 11th layer deposition for the case of type 3 and 5 paths of ADDS, as shown in Figure 13. The residual stress abruptly increases from the first layer to the 3rd layer deposition when the type 8 path of UDDS is adopted, while that steadily increases up to the 11th layer deposition when the type 3 and 5 paths of ADDS are applied. The fluctuation of the residual stress at the location of the maximum stress slightly increases with an increasing number of deposited layers for the case of the type 8 path of UDDS, whereas that decreases with an increasing number of deposited layers for the case of type 3 and 5 paths for ADDS, as shown in Figure 13. The fluctuation of the residual stress is significantly reduced from the deposition of the 11th layer when the type 3 and 5 paths for ADDS are adopted. In addition, similar residual stress distributions are repeated from the 11th layer deposition to the 17th layer deposition before cooling, as shown in Figure 14. From these results, it is revealed that the steady-state from the viewpoint of the residual stress is formed from the deposition of the 11th layer when the type 3 and 5 paths of ADDS are used.

The number of deposited layers for the steady-state from the viewpoint of the residual stress formation is identical to that from the viewpoint of the heat transfer phenomenon. From this result, it is revealed that a stable residual stress state is formed in the fabricated part when the steady-state is reached in terms of the heat transfer phenomenon. Values of effective and first principal stresses at the location of the maximum stress for the type 3 path of ADDS are greater than those for the type 5 path of ADDS by nearly 0–8% and 1–23%, respectively, as shown in Figure 13. From this result, it is considered that the possibility of cracking in the fabricated part can be reduced when the type 5 path of ADDS is applied. Based on the above results, a suitable deposition strategy is determined to be the type 5 path of ADDS.

### 3.4. Preheating Temperature

**Figure 15:**
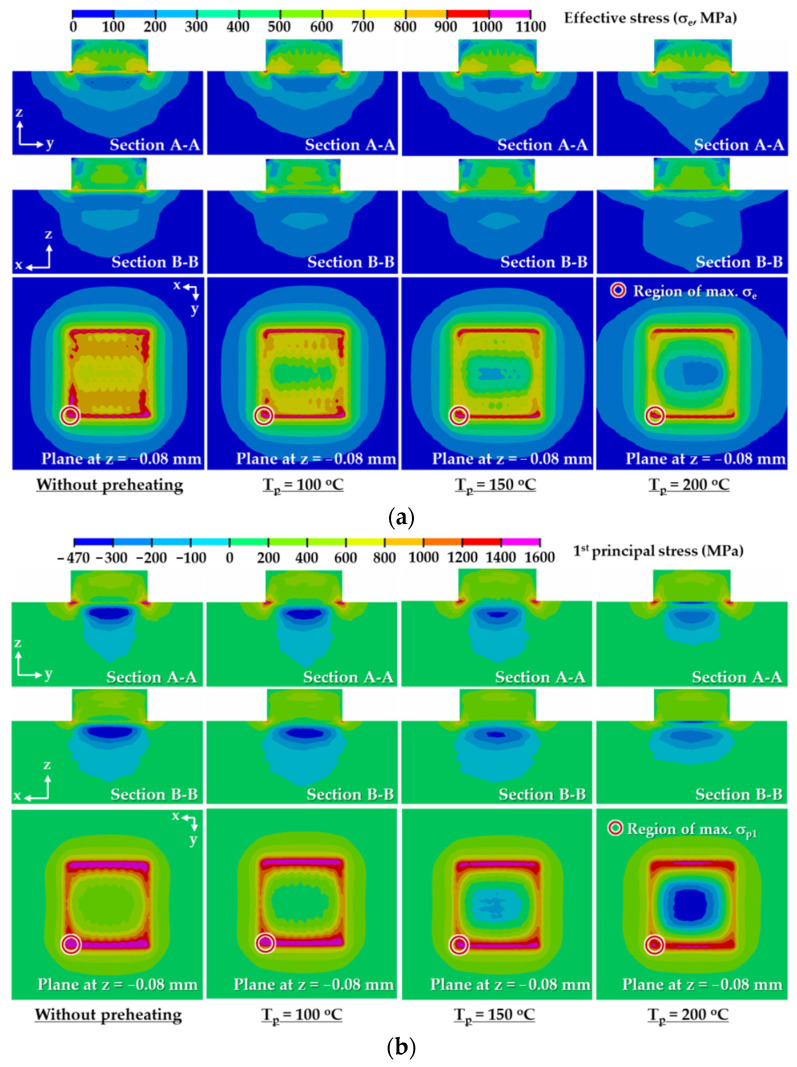
Residual stress distributions for different preheating temperatures (after the completion of cooling): (**a**) effective stress distributions; (**b**) first principal stress distributions.

**Figure 16:**
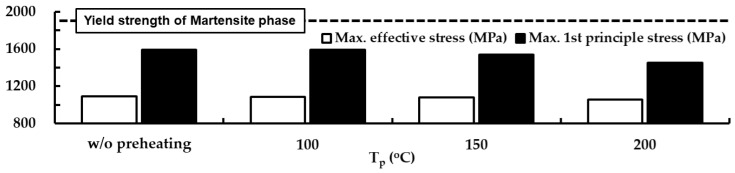
Influence of the preheating temperature on maximum effective and first principle stress after completion of cooling.

FEAs for different preheating temperatures are carried out to estimate an appropriate preheating temperature using the determined suitable deposition strategy. Figure 15 shows the influence of the preheating temperature on residual stress distributions in the designed FE model after the completion of cooling. A highly stressed region, marked in red and magenta colors in Figure 15a,b, significantly decreases when the preheating temperature increases. This is because the compressive region, where the compressive residual stress occurs, augments in the vicinity of the boundary between the deposited region and the substrate when the preheating temperature increases, as shown in Figure 15b. The compressive region in the vicinity of the boundary between the deposited region and the substrate gradually expands from the center to the edge of the highly stressed region when the preheating temperature increases. The compressive region under the deposited region decreases when the preheating temperature augments. The location of the maximum residual stress is always identical irrespective of the preheating temperature. The maximum values of the effective and first principal stresses decrease when the preheating temperature increases, as shown in Figure 16. When the preheating temperature increases to 200 °C, the maximum values of the effective and first principal stresses decrease by nearly 3% and 9%, respectively. These are due to the fact that the tensile stress in the highly stressed region is reduced by the increased compressive stress in the vicinity of the boundary between the deposited region and the substrate.

The results of the phase analysis in the FEAs predict that martensite dominantly appears in the location of the maximum stress after the completion of cooling. The final temperatures of the FE model after the completion of cooling ranges lie in the range of 25–27 °C. The yield strength of the martensite phase for AISI 1045 in the range of the final temperature is estimated to be nearly 1900 MPa, as shown in Figure 4e. Estimated maximum effective and first principal stresses for applied preheating temperatures lie in ranges of 1057–1090 MPa and 1449–1593 MPa, respectively, as shown in Figure 16. Comparing the yield strength with the estimated residual stress, it is shown that yielding does not occur in the FE model at the preheating temperatures applied to the FEAs.

**Figure 17:**
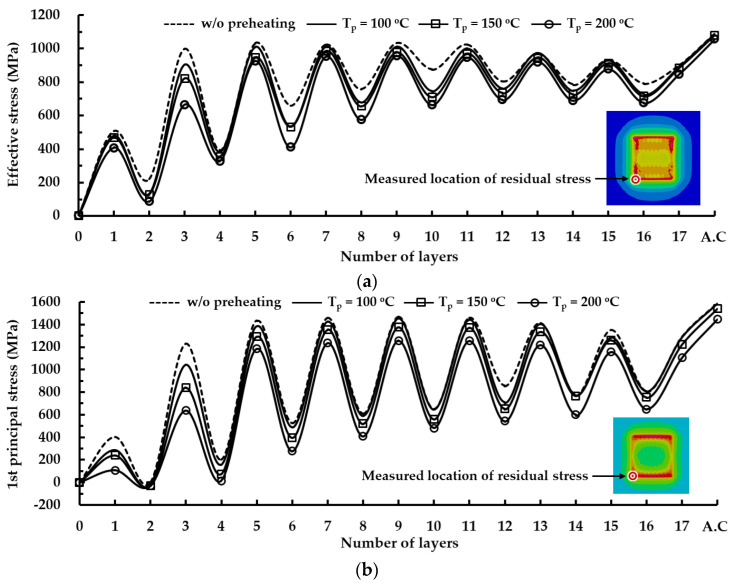
Histories of residual stresses at the location of maximum stress for different preheating temperatures: (**a**) effective stress; (**b**) first principal stress.

**Figure 18:**
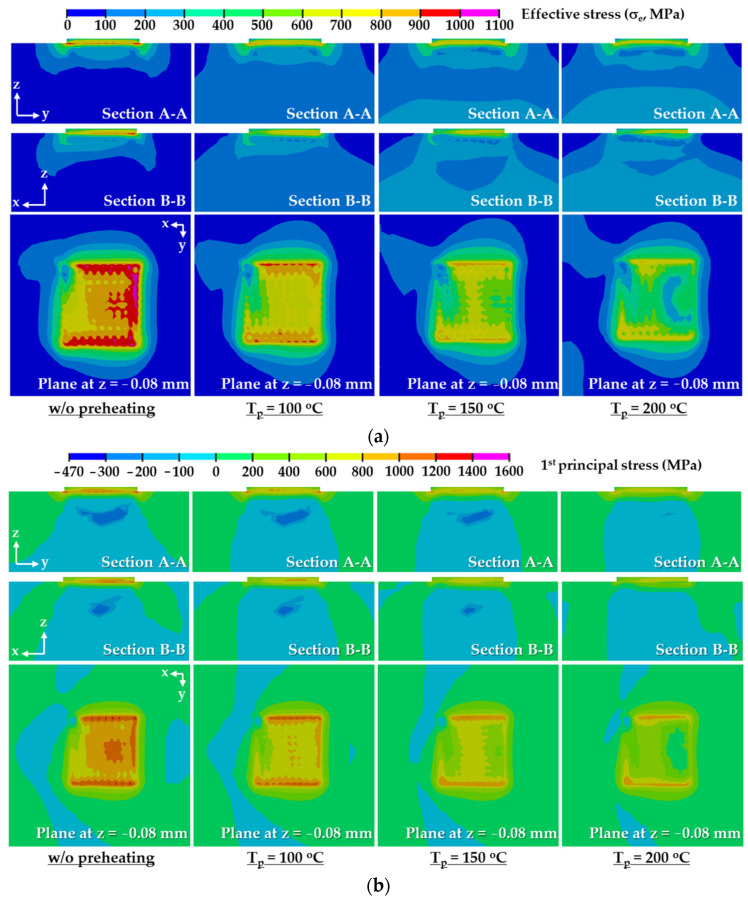
Residual stress for different preheating temperatures (after completion of the 3rd layer deposition): (**a**) effective stress distributions; (**b**) first principal stress distributions.

**Figure 19:**
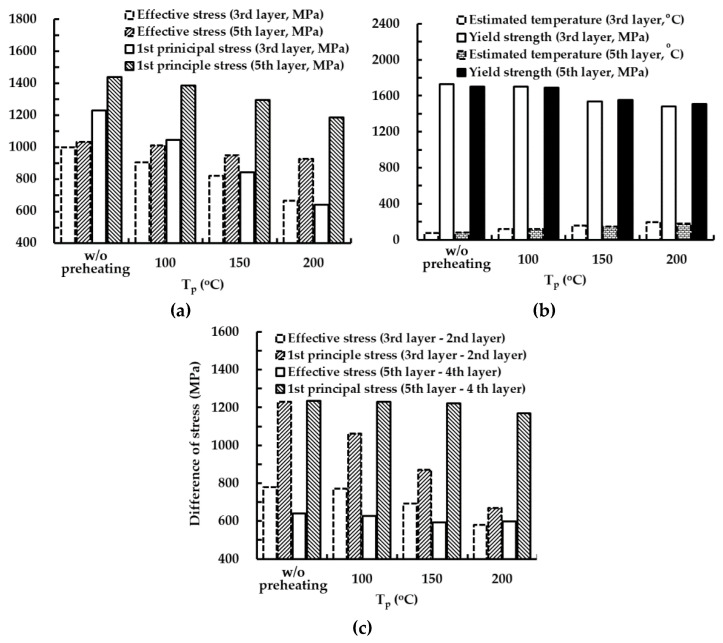
Effects of preheating temperatures on residual stress and yield strength corresponding to the estimated temperature at the location of the maximum stress for a low layer deposition: (**a**) residual stresses; (**b**) yield strengths corresponding to the estimated temperature; (**c**) difference of residual stress.

Figure 17 shows histories of residual stresses at the location of the maximum stress for different preheating temperatures. Effective and first principal stresses decrease with increasing preheating temperature. The residual stress significantly decreases during the deposition of a low layer when preheating is applied to the substrate, as shown in Figure 17, Figure 18 and Figure 19a. The reduction of the residual stress is noticeable after the deposition of the 3rd layer when the preheating of the substrate is adopted. Through preheating of the substrate up to 200 °C, effective and first principal stresses at the location of the maximum stress after the completion of the 3rd layer deposition can decrease by nearly 33% and 48%, respectively. The sudden increase in the residual stress, which occurred during low layer deposition without preheating of the substrate, is greatly relieved when preheating of the substrate is applied. From these results, it is revealed that a smooth transition of the residual stress during successive depositions can be induced by increasing the preheating temperature of the substrate.

From the results of the phase analysis in the FEAs, it is noted that the martensite phase dominantly appears in the highly stressed region from low layer deposition. Using the relationship between the temperature and the yield strength for the martensite phase of AISI 1045, yield strengths corresponding to temperatures at the location of the maximum stress after the completion of the cooling stage are estimated, as shown in Figure 19b. Comparing the residual stresses and the estimated yield strengths, it is shown that the yielding phenomenon does not take place in the designed FE model irrespective of preheating of the substrate.

**Figure 20:**
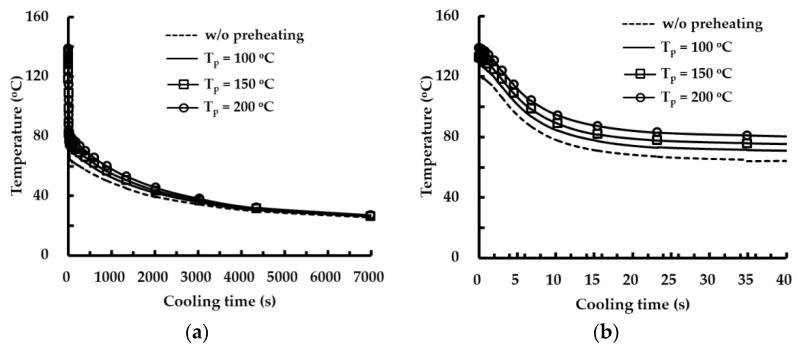
Cooling time–temperature curves for different preheating temperatures: (**a**) cooling time–temperature curves for total cooling time; (**b**) cooling time–temperature curves up to 40 s.

The fluctuation of the histories of the residual stress decreases when the preheating temperature increases, as shown in Figure 17. The difference of the residual stress between the depositions of successive layers is reduced when the preheating of the substrate is adopted, as shown in Figure 17 and Figure 19c. The reduction of the difference of the residual stress between successive layers decreases the deviation of the strain of the corresponding region. In addition, the reduced strain deviation can contribute to decreasing the possibility of cracking in the vicinity of the deposited region. From these results, it is considered that the possibility of fatigue cracking can be remarkably reduced for the case of a large volume deposition when preheating of the substrate is adopted. The difference in the residual stress significantly decreases with increasing the preheating temperature during low layer deposition. The difference of the residual stress between the deposition of the 3rd layer and that of the second layer is noticeably reduced when preheating is applied to the substrate. Through preheating of the substrate up to 200 °C, differences in the effective and first principal stresses between the deposition of the 3rd layer and that of the second layer at the location of the maximum stress decrease by nearly 26% and 46%, respectively. From these results, it is elucidated that the possibility of fatigue cracking and sudden excessive deformation in low layer deposition can remarkably decrease when the preheating temperature of the substrate increases. Figure 20 shows cooling time–temperature curves at the location of the maximum stress for different preheating temperatures. The temperature after the completion of the deposition is estimated to be nearly 120 °C when preheating is not applied to a substrate. The increment of temperature after the completion of the deposition is less than 19 °C when the preheating temperature of the substrate is increased up to 200 °C. The rapid decrease in the temperature takes place within a cooling time of 20 s, as shown in Figure 20. The temperature at the location of the maximum stress is nearly 82 °C when the preheating temperature and the cooling time are 200 °C and 20 s, respectively. The cooling curves for different preheating temperatures are similar exclusive of a small offset of the temperature between cooling time–temperature curves in the rapid cooling region. The maximum temperature offset is less than 16 °C when the preheating temperature and the cooling time are 200 °C and 20 s, respectively. From these results, it is revealed that the adopted preheating temperature of the FEAs hardly affects the temperature distribution after the completion of the deposition and the cooling rate of the cooling stage. In addition, it is considered that an additional increment of the thermal deformation of the fixture and the base plate of the hybrid system is negligible when the preheating temperature specified for the FEA is applied to the substrate.

Based on the above results, an appropriate preheating temperature of the substrate is determined to be 200 °C.

## 4. Conclusions

In this paper, the effects of the deposition strategy and the preheating temperature on thermomechanical characteristics of Inconel 718 super-alloy deposited on an AISI 1045 substrate using a DED process were investigated through FEAs. The FE model was developed to predict the temperature and residual stress distributions. Temperature-dependent thermomechanical properties considering the phase transformation were used to properly estimate thermomechanical behaviors during the deposition and the cooling.

The coefficient of the heat sink was estimated from a comparison of temperature histories of experiments and those of FEAs to obtain appropriate FE models. A suitable coefficient of the heat sink was chosen as 1000 W/m °C. The results of FEAs using the suitable coefficient of the heat sink were compared to those of experiments from the viewpoint of the temperature history. From the results of the comparison, it was shown that the FE model with the selected suitable coefficient of the heat sink could properly estimate thermal characteristics during the deposition and the cooling.

The influence of deposition strategies on residual stress distributions was examined via FEAs for a small volume deposition with two layers. The cross-sectional residual stress distribution of the ESRs formed almost symmetrically concerning the centerline of the deposited bead for ADDSs, while that appeared asymmetrically for UDDSs. The combination of the deposition direction and angle between successive layers greatly affected the residual stress distribution and the critical location. The maximum effective stress after the completion of cooling was minimized for cases of type 3 and 5 paths for ADDSs, and type 8 paths for UDDSs. From the results of FEAs for a small volume deposition, type 2 and 5 paths of ADDSs and type 8 paths of UDDSs were chosen as feasible deposition strategies.

The effects of the deposition strategy on residual stress distributions and histories were investigated through FEAs for large volume deposition. Unlike the UDDS, the effective stress distributions of the ADDS appeared almost symmetrical to reference axes of the model. The residual stress of the type 8 path for UDDS was greater than that of type 3 and 5 paths for ADDS. The maximum effective and first principal stresses after the completion of cooling for UDDS were greater than those for ADDS by nearly 4% and 11%, respectively. The fluctuation of the residual stress at the location of the maximum stress slightly increased with an increasing number of deposited layers for the case of the type 8 path of UDDS, whereas that decreased with an increasing number of deposited layers for the case of type 3 and 5 paths for ADDS. Values of the effective and first principal stresses at the location of the maximum stress for the type 3 path of ADDS were greater than those for the type 5 path of ADDS by nearly 0–8% and 1–23%, respectively. Based on the above results, a suitable deposition strategy was determined to be the type 5 path of ADDS.

The effects of the preheating temperature of the substrate on residual stress distribution during deposition and cooling were examined using the suitable deposition strategy. When the preheating temperature was increased to 200 °C, the effective and first principal stresses decreased by nearly 33% and 48%, respectively. The sudden increase in the residual stress, which occurred during low layer deposition without preheating of the substrate, was greatly relieved when preheating of the substrate was applied. The fluctuation and the difference of the residual stress between the depositions of successive layers were significantly reduced when preheating of the substrate was adopted. Based on the above results, an appropriate preheating temperature of the substrate was determined to be 200 °C.

Further experiments and FEAs should be performed to obtain optimal deposition strategy and preheating temperature for the sake of improving thermomechanical characteristics in the vicinity of the deposited region. In addition, additional experiments to measure the residual stress in the vicinity of the deposited region are needed to develop the improved FE model.

## Figures and Tables

**Table 1:** Characteristic dimensions of FE models.

Initial Bead Width (mm)	Bead Thickness (t_b_, mm)		Hatching Distance (mm)	Height of Deposited Region (mm)	
	1st Layer	2nd–17th Layer		2 Layers	17 Layers
0.78	0.135	0.25	0.50	0.385	4.135

**Table 2:** Conditions for deposition experiments and FEAs.

Radius of Laser Beam (r_e_, mm)	Power of Laser (P_L_, mm)	Scan Speed of Laser (V, mm/min)	Feed Rate of Powders (F, g/min)	Flow Rate of Shield Gas (*L*/min)	Penetration Depth (δ, mm)	Radius at z Coordinate (r(z))	Preheating Temperature (T_p_, °C)
0.5	350	1000	≈10.3	≈7.0	=t_b_	≈r_e_	100–200

**Table 3:** Chemical composition of Inconel 718 (wt %) [39].

Ni	Cr	Fe	Mo	Si	Mn	C	Co	Cu	Others
55	21	Balance	3.3	0.35	0.35	0.08	1.0	0.3	<5.0

**Table 4:** Chemical composition of AISI 1045 (wt %) (cited mill test certificate for the used AISI 1045).

Fe	Ni	Cr	Si	Mn	C	Cu	Others
Balance	0.005	0.015	0.247	0.757	0.444	0.01	<0.1

## Data Availability

Data sharing is not applicable to this article.

## Abbreviations

DED	Directed energy deposition
LENS	Laser engineered net shaping
FE	Finite element
FEA	Finite element analysis
TC	Thermocouple
ML	Measured location
TD	Thickness direction
ESR	Excessively stressed region
ADDS	Alternative directional deposition strategy
UDDS	Unidirectional deposition strategy
FDS	Feasible deposition strategy
A.C	After cooling
